# Development of a Method for Rapid Determination of Morpholine in Juices and Drugs by Gas Chromatography-Mass Spectrometry

**DOI:** 10.1155/2018/9670481

**Published:** 2018-04-26

**Authors:** Mengsi Cao, Pingping Zhang, Yanru Feng, Huayin Zhang, Huaijiao Zhu, Kaoqi Lian, Weijun Kang

**Affiliations:** ^1^School of Public Health, Hebei Medical University, Shijiazhuang 050017, China; ^2^Department of Reproductive Genetic Family, Hebei General Hospital, Shijiazhuang 050017, China; ^3^Hebei Province Key Laboratory of Environment and Human Health, Shijiazhuang 050017, China

## Abstract

A reliable derivatization method has been developed to detect and quantify morpholine in apple juices and ibuprofen with gas chromatography-mass spectrometry. Morpholine can react with sodium nitrite under acidic condition to produce stable and volatile N-nitrosomorpholine derivative. In this experiment, various factors affecting the derivatization and extraction process were optimized, including volume and concentration of hydrochloric acid, quantity of sodium nitrite, derivatization temperature, derivatization time, extraction reagents, and extraction time. The derivative was extracted with dichloromethane and determined by gas chromatography-mass spectrometry. The linearity range of morpholine was 10–500 *μ*g·L^−1^ with good correlation, and limits of detection (LOD) and limits of quantification (LOQ) were 7.3 *μ*g·L^−1^ and 24.4 *μ*g·L^−1^, respectively. Low, medium, and high concentrations of morpholine were added in apple juices and ibuprofen samples to evaluate standard recovery rate and relative standard deviation. The spiked recovery rate ranged from 94.3% to 109.0%, and the intraday repeatability and interday reproducibility were 2.0%–4.4% and 3.3%–7.0%, respectively. The developed method has good accuracy and precision. This quantitative method for morpholine is simple, sensitive, rapid, and low cost and can successfully be applied to analyze the residual morpholine in apple juices and drug samples.

## 1. Introduction

Morpholine (tetrahydro-2H-1,4-oxazine), a heterocyclic secondary amine, is a colorless, hygroscopic, alkaline, oily liquid at normal temperature and pressure with an ammoniacal odor and is miscible with water and organic solvents in any ratio [1]. Morpholine is used as an emulsifier for protective wax coating on apples and other fruits to keep them fresh and storable [2–4]. Nowadays, more and more people like to drink fresh juice instead of fresh fruit, and some manufacturers produce fresh juice together with the pericarp to improve dietary fiber in fruit juice and economic benefits, thereby increasing the residual content of morpholine in the juice, such as apple juice. The compound also effectively suppresses the hatching process of the eggs of golden apple snails, a known pest of the rice crops in Asia, and thereby controls the reproduction of those snails to protect the rice crops [5]. Being a cyclic amine, morpholine is commonly used in pharmaceutical industries for synthesis of different active pharmaceutical substances, such as morinidazole [6], and to increase aqueous solubility of gefitinib [7]. Morpholine has been used for preparing a series of new antimicrobial and antiviral diphenyl diselenides [8]. It is also used as a reagent to prepare the morpholine derivative, 4-(2-aminoethyl) morpholine, also called AEM [9]. AME, triethylamine, and methacryloyl chloride are used to synthesize N-ethyl morpholine methacrylamide (EMA) [10]. EMA is a pH-sensitive polymer hydrogel which is used to prevent crystallization of ibuprofen [11]. Consequently, morpholine residues may be present in the production of ibuprofen. Morpholine causes irritation of eye, skin, and digestive tract and may be absorbed in the body through skin contact, inhalation, and ingestion [1]. As a result, the use of morpholine has been prohibited as an emulsifier in protective wax coating on citrus fruits, apples, and cosmetic preparations in the European Union (EU) [1, 12]. As per Health Canada Monograph [13], the no-observed-adverse-effect level (NOAEL) of morpholine is 96 mg·kg^−1^ of body weight (bw) day^−1^ and the acceptable daily intake (ADI) is 0.48 mg·kg^−1^ of bw day^−1^ [14]. Therefore, establishing a rapid and effective method to detect and quantify morpholine in fruit juices and pharmaceuticals is of primary importance.

In recent years, numerous studies have reported various analytical methods for qualitative and quantitative estimation of morpholine. These analytical methods employed various available analytical techniques, such as gas chromatography (GC) [15–17], gas chromatography-mass spectrometry (GC-MS) [18], gas-liquid chromatography-high resolution mass spectrometry (GLC-MS) [19], liquid chromatography (LC) [20], ultra performance liquid chromatography (UPLC) [21], hydrophilic interaction liquid chromatography with electrospray ionization and tandem mass spectrometry (HILIC-ESI-MS/MS) [22], and ultrahigh performance liquid chromatography-high resolution mass spectrometry (UHPLC-HRMS) [14]. However, these published methods have different disadvantages, such as tedious operation steps [14] and high cost [22]. Applying the derivatization method with 2,4-dinitrofluorobenzene (2,4-DNFB) by GC-MS to detect morpholine has better sensitivity, but has low stability [18].

This experiment was based on some of the secondary amines that could react with sodium nitrite to produce volatile N-nitrosamines (NAms) under acidic conditions [23]. We found that morpholine as a cyclic secondary amine can generate N-nitrosomorpholine (NMOR) by using sodium nitrite as the derivatization reagent under acidic condition, and NMOR which is stable and volatile can be determined by GC-MS. Our team used to establish a method to determine ketamine in urine and plasma by this derivative method and obtained good experimental results [24]. We have extensive experience about this derivatization reaction. Therefore, various factors affecting derivatization process and extraction efficiency can be optimized to develop a reliable method for rapid determination of morpholine in apple juice and drug granules through GC-MS. Compared with other existing derivatization methods, sodium nitrite and hydrochloric acid as derivatization reagents are cheap and obtained easily in this experiment. The samples only needed centrifugate and filter without complicated sample pretreatment process and was analysed rapidly by GC-MS. The consumption of organic solvents was very small in the whole test process, thereby reducing the pollution of the environment. This study established a rapid, sensitive, simple, low-cost, and reliable method to determine morpholine in apple juice and drugs, and had highly realistic application value.

## 2. Experimental

### 2.1. Chemicals

All chemicals and reagents were of analytical grade unless otherwise stated. Standard morpholine was purchased from Aladdin Reagent Co., Ltd. (Shanghai, China). The derivatization reagents of sodium nitrite (NaNO_2_) and hydrochloric acid (HCl) were purchased from Henan Jiaozuo Three Chemical Plant (Jiaozuo, China) and Shijiazhuang Reagent Factory (Shijiazhuang, China), respectively. Dichloromethane, ethyl acetate, chloroform, n-hexane, and carbon disulfide from Xilong Chemical Factory (Shantou, China) or Tianjin General Chemical Reagent Factory (Tianjin, China) were tested to select the most optimal extraction reagent. Pure water (18.2 M*Ω*/cm) was obtained from Heal Force SMART-N ultrapure water system (Hong Kong).

### 2.2. Quantitative Methods and Quality Control Samples

Stock standard solution of morpholine (50 mg·L^−1^) was prepared in pure water. Working calibrators at 10, 25, 50, 100, 200, 300, 400, and 500 *µ*g·L^−1^ were prepared by diluting in pure water, and the calibration curve was fitted by linear regression method through the measurement of the peak areas corresponding to the concentrations. The acceptance criterion for the calibration curve is a correlation coefficient of 0.99 or better. Quality control (QC) samples were prepared by freshly spiking the appropriate working solution into blank apple juice and ibuprofen samples to prepare concentrations of 50, 200, and 400 *μ*g·L^−1^ for morpholine. The series of standard solution and QC samples were freshly prepared before use.

### 2.3. Pretreatment of Samples

The apple juices were obtained from a local supermarket and filtered with 0.22 *µ*m membrane filter. Ibuprofen granules were purchased from a local pharmacy and dissolved in purified water and centrifuged (10,000 rpm for 15 min) after mixing. The supernatant liquid was filtered with 0.22 *µ*m membrane filter. All the samples were stored at 4°C.

### 2.4. Derivatization and Liquid-Liquid Extraction

A certain amount of morpholine stock standard solution was added to 20 mL of apple juice or ibuprofen solution in a 50 mL disposable sample pretreatment tube. The samples were centrifuged and filtered as described in the Section 2.3. To 2.0 mL of pretreated apple juice or ibuprofen solution, 200 *μ*L of 0.05 mol·L^−1^ HCl and 200 *μ*L of saturated NaNO_2_ were added and vortex-mixed. The resultant solution was placed in a 10 mL glass test tube and mixed thoroughly. The mixture was heated at 40°C for 5 min on a heating block. After cooling, 0.5 mL of dichloromethane was added, and the mixture was vortex-mixed for 1 min and allowed to stand for 10 min to extract the derivative. Then, 200 *μ*L of organic layer was transferred with a micropipette to a tipped glass tube and placed in an ice bath to prevent the volatilization of dichloromethane and the impact on experiment results. Then, 1 *μ*L of this organic layer was injected into the GC-MS with a 10 *μ*L syringe (from Agilent).

### 2.5. GC-MS Analysis

An Agilent Technologies (Little Falls, DE, USA) gas chromatograph 7890 equipped with an electronically controlled split/splitless injection port, an inert 5975C mass selective detector with electron impact (EI) ionization chamber, and a 7683B series injector/autosampler were employed for identification and quantification of N-nitrosomorpholine that was the derivative of morpholine.

The GC separation was conducted with a TM-1701 30 m × 0.32 mm I.D., 0.5 *μ*m film thickness column (Techcomp, China). The carrier gas was helium with a constant flow rate of 2 mL·min^−1^. The injection volume was 1 *μ*L and was vaporized at 250°C with a 1 : 7 split ratio. The GC oven was operated with the following temperature program: initial temperature 100°C held for 4 min and programmed to 120°C at a rate of 10°C min^−1^ and held for 3 min, and then ramped at 20°C min^−1^ –250°C and held for 5 min. The total run time was 18 min.

Two different ions were selected to detect and quantify N-nitrosomorpholine (86.1, 116.1) at the selected ion-monitoring (SIM) mode. Ionization was performed by electron impact (EI) mode at 70 eV energy. The temperatures used were 280°C for the transfer line, 230°C for the ion source, and 150°C for the MS quadrupole. The solvent delay was 4.5 min.

## 3. Results and Discussion

### 3.1. Principles of Derivatization and Identification of Derivative

**Figure 1:**
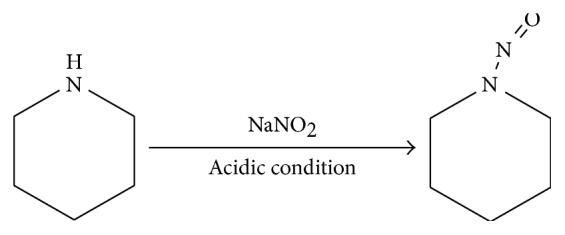
The derivatization reaction of morpholine.

**Figure 2:**
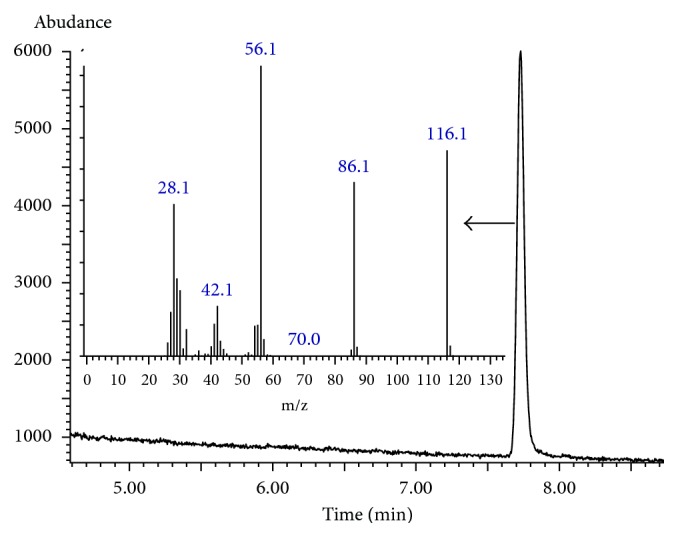
The total ion current chromatogram and mass spectra of the N-nitrosomorpholine.

Morpholine, as a secondary amine, reacts with sodium nitrite under acidic conditions to produce stable and volatile NMOR which can be determined by GC-MS. The reaction is shown in Figure 1. 2.0 mL of 400 *μ*g·L^−1^ morpholine standard solution was used to verify the derivatization reaction. The total ion current chromatogram and mass spectra of the NMOR derivative are shown in Figure 2. Analyses of mass spectra and MS data of the derived sample proved that the derivative was NMOR.

### 3.2. Optimization of Derivatization and Extraction

A rapid and low-cost derivatization technique has been developed for detection and determination of morpholine. The derivatization process of morpholine has been described in the Section 2.4. Various factors associated with derivatization and extraction process were optimized, which included concentration and dosage of hydrochloric acid (HCl), the amount of saturated sodium nitrite (NaNO_2_), derivatization temperature, derivatization time, the extraction reagents, and extraction time.

#### 3.2.1. Concentration and Quantity of Hydrochloric Acid

**Figure 3:**
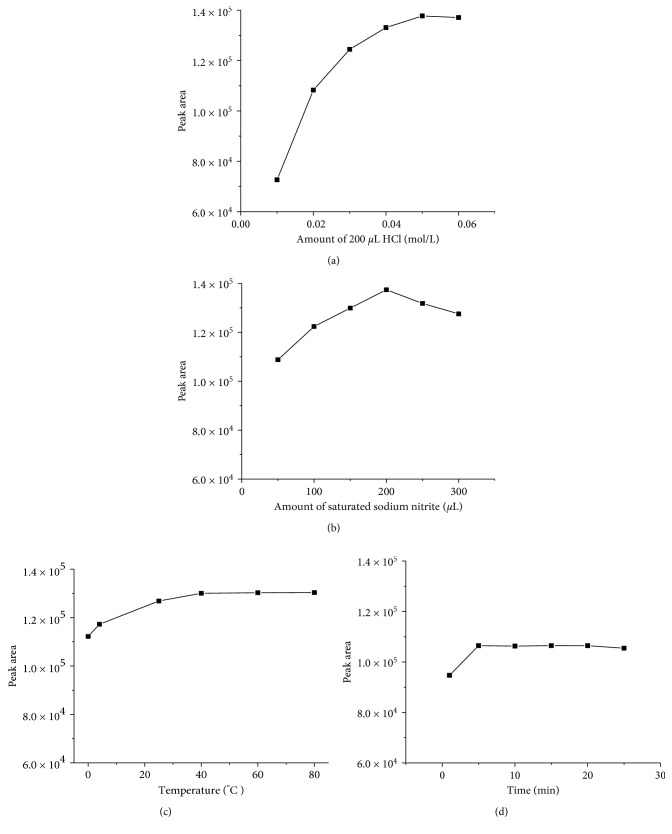
The effects of hydrochloric acid concentration and quantity (a), the amount of saturation solution of sodium nitrite (b), derivative reaction temperature (c), and time (d).

The derivatization process was affected by the concentration and quantity of hydrochloric acid. The concentration of HCl was optimized as the first step. The effects of adding 200 *μ*L of HCl with different concentrations between 0.01 and 0.06 mol·L^−1^ are shown in Figure 3. The derivatization rate was found to increase with the increasing concentration of HCl in the range from 0.01 to 0.05 mol·L^−1^ and then became stable. Thus, the best result was obtained when 200 *μ*L of 0.06 mol·L^−1^ HCl was added during the process of derivatization.

#### 3.2.2. The Amount of Saturation Solution of Sodium Nitrite

Optimum quantity of saturation solution of sodium nitrite required for the derivatization process was determined (Figure 3) by varying the addition of saturation solution of sodium nitrite in the range of 50–300 *μ*L. The derivatization yields increased with the addition of saturation solution of sodium nitrite up to 200 *μ*L and then became stable. Therefore, the optimum volume of saturation solution of sodium nitrite for derivatization was 200 *μ*L.

#### 3.2.3. Derivatization Temperature and Derivatization Time

The effects of derivatization temperature and time were tested in this experiment. The effect of temperature (0°C (ice-bath), 4°C (refrigeration), 25°C (room temperature), 40°C, 60°C, and 80°C) on derivatization was investigated. The rate of derivatization increased with reaction temperature and then became stable at 40°C (Figure 3). Therefore, 40°C was selected as the optimum temperature for this experiment. Moreover, the effect of reaction time on derivatization process was investigated; the reaction time was varied between 1 and 30 min. The derivatization leveled off at 5 min (Figure 3), suggesting the optimum reaction time to be 5 min.

#### 3.2.4. Extraction Reagents and Extraction Time

**Figure 4:**
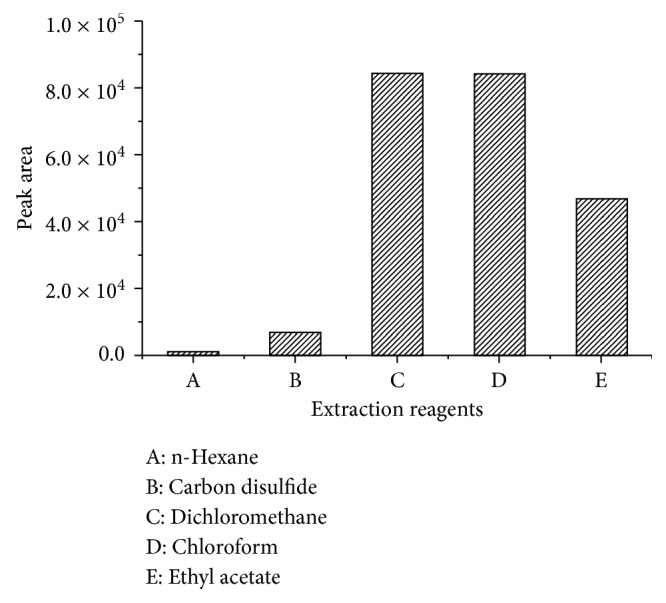
Extraction effects of different extraction reagents.

Selection of suitable solvent is an important criterion for extraction of the derivative. The extraction efficiencies of n-hexane, dichloromethane, chloroform, carbon disulfide, and ethyl acetate were evaluated as shown in Figure 4. The study revealed that dichloromethane and chloroform afforded optimum extraction of the derivative. Finally, dichloromethane was selected as the extraction reagent. 2.0 mL of dichloromethane was used to extract the derivative, and 1.5 mL organic layer was transferred to a tipped glass tube and dried with a slow stream of nitrogen at room temperature. The dried substances were dissolved in 100 *µ*L ethyl acetate before GC analysis. However, the experiment resulted in poor precision as indefinite derivative was blown away in the nitrogen blowing process. To improve the extraction efficiency and stabilization, 0.5 mL dichloromethane was added and vortex mixed for 1 min followed by standing for 10 min to extract the derivative.

**Figure 5:**
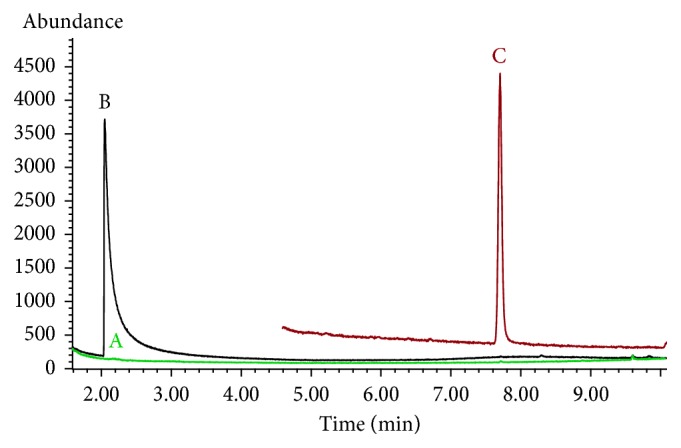
The total ion current chromatograms of morpholine resulting from different methods: direct detection of 400 *μ*g·L^−1^ (A) and 20 mg·L^−1^ (B) morpholine prepared in dichloromethane and detection of 400 *μ*g·L^−1^ morpholine prepared in pure water after the proposed derivatization (C).

An attempt to detect morpholine (400 *μ*g·L^−1^) by GC-MS without any derivatization process failed, as the method could not detect any signal of the compound (Figure 5). In another attempt, morpholine produced similar signal abundance in two different samples (20 mg·L^−1^ in dichloromethane without derivatization and 400 *μ*g·L^−1^ in pure water after derivatization) (Figures 5 and 5). The proposed derivatization method was about 65 times more sensitive than the direct detection. Kataoka [16] had compared the effects of commonly used derivatization reagents, such as acylation, silylation, dinitrophenylation, permethylation, carbamate formation, sulfonamide formation, and phosphoamide formation for analysis of secondary amines by GC. However, sodium nitrite and hydrochloric acid are preferred as derivatization agents, since they are cheaper and easily available compared to other reagents. Sacher et al. [18] established a method based on derivatization of the amines with benzenesulfonyl chloride. However, usage of many reagents and long derivatization process (1 hour) and operation time (morpholine peak appeared at 18 min) made this method practically inconvenient. In comparison, the proposed method involves less derivatization process (5 min) and operation time (morpholine peak appeared at 7.72 min).

The proposed method has many advantages compared to previously published methods [14, 15, 22] (Table 1). Use of MS detector in this study produced similar sensitivity as HILIC-ESI-MS/MS [22], and both the methods yielded better results than flame ionization detection (FID) [15]. Dawei Chen et al. [14] established a reliable method to determine morpholine residues by UHPLC-HRMS combined with dispersive micro-solid-phase extraction (DMSPE). The method is more sensitive but involves complicated sample pretreatment process and costly instrumentation.

### 3.3. Selection of Chromatographic Column

In the preliminary experiment, HP-5 nonpolar chromatographic column (dimensions: 30 m × 0.32 mm × 0.25 *µ*m; stationary phase: 5% phenyl-95% methylpolysiloxane) and TM-1701 medium polarity chromatographic column (dimensions: 30 m × 0.32 mm × 0.5 *μ*m; stationary phase: 14% cyanopropyl phenyl-86% dimethyl polysiloxane) were employed. HP-5 column resulted in peak tailing of peak and high baseline. TM-1701 column provided better peak shape under the optimized conditions and therefore suited the experiment.

### 3.4. Method Validation

#### 3.4.1. Linearity, Detection Limit, and Quantitative Limit

Calibration curve was constructed by plotting the peak area against the concentration range from 10 to 500 *µ*g·L^−1^ of morpholine. The linear regression equation was *A* = 471.2*c* − 2263.8, in which *c* corresponds the concentrations and *A* corresponds the peak areas. The results obtained a good linearity of the analytical range which was 10–500 *µ*g·L^−1^ with the coefficient of determination (*R*^2^) of the calibration curve for morpholine higher than 0.999. In this method, the limit of detection (LOD) and the limit of quantification (LOQ) were calculated as 3 and 10 times the *S*/*N* ratio, which indicated 7.3 *µ*g·L^−1^ and 24.4 *µ*g·L^−1^, respectively.

#### 3.4.2. Accuracy and Precision

Through adding standard solution of morpholine with high concentration to the apple juice and ibuprofen blank samples, three different spiked samples with final concentration levels of 50, 200, and 400 *µ*g·L^−1^ were obtained. The samples with each concentration level were determined on six times a day over three consecutive days. The spiked recovery rate, intraday repeatability, and interday reproducibility were 94.3%–109%, 2.3%–4.4%, and 4.8%–5.2% for apple juice spiked samples, and 96%–107.9%, 2%–4.4%, 3.3%–7% for ibuprofen spiked samples, respectively (Table 2). The results indicated that the method was suitable for determining morpholine with favourable accuracy and precision.

### 3.5. Application to Real Samples

**Figure 6:**
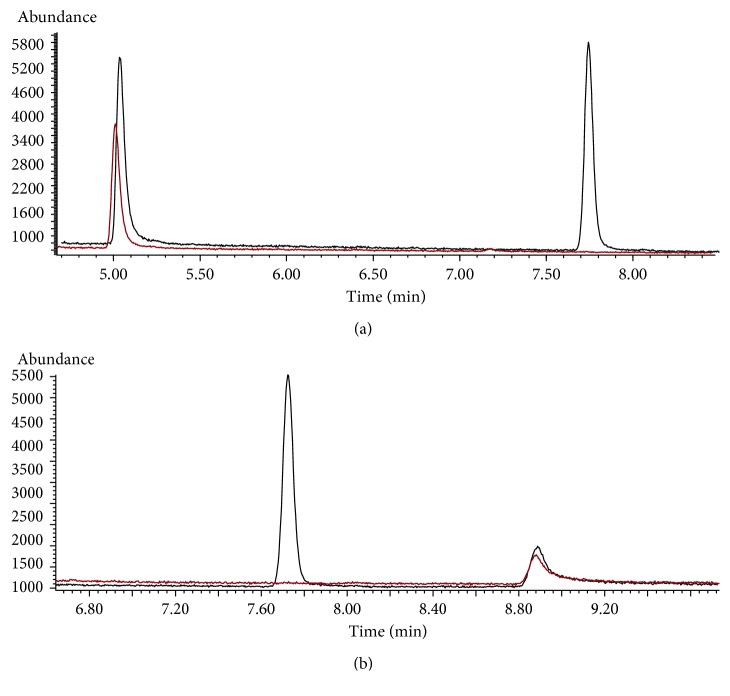
The total ion current chromatograms of apple juice samples and spiked samples (400 *μ*g·L^−1^) (a) and ibuprofen samples and spiked samples (400 *μ*g·L^−1^) (b).

Samples of apple juice and ibuprofen granules were analysed by this method under optimal conditions using standard addition method. However, morpholine was not detected in any real samples. Thus, using the standard addition method, morpholine was detected in apple juice and ibuprofen granules samples. The total ion current chromatograms of the real samples of apple juice and ibuprofen granules and their spiked samples (400 *μ*g·L^−1^) are shown in Figure 6.

## 4. Conclusions

According to that morpholine could react with sodium nitrite to generate the stable and volatile N-nitrosomorpholine under acidic conditions, we established a rapid, sensitive, simple, low-cost, and reliable method to detect morpholine in apple juices and drugs. This method had been successfully analysed of spiked samples with low detection limit and favourable accuracy and precision. It can provide technical support to establish the national standards of morpholine in fruit juices and pharmaceuticals and monitor the residue of morpholine in the future.

## Figures and Tables

**Table 1:** Comparison of the proposed method with previously published methods.

Sample	The test process of sample			LOQ	Reference
	Sample pretreatment	Derivatization reaction	Determination		
Apple juice and ibuprofen	Centrifugation and filtration	Sodium nitrite under acidic condition	Gas chromatography-mass spectrometry (GC-MS)	24.4 *μ*g·L^−1^	This work
Steam condensate	—	—	Chromatography with multimode inlet and flame ionization detection (GC-MI-FID)	100 *μ*g·L^−1^	[15]
Citrus and apples	15 mL 1% acetic acid in methanol	—	Hydrophilic interaction liquid chromatography with electrospray ionization and tandem mass spectrometry (HILIC-ESI-MS/MS)	10 *μ*g·kg^−1^	[22]
Citrus and apples	Dispersive micro-solid-phase extraction (DMSPE)	—	Ultrahigh performance liquid chromatography-high resolution mass spectrometry (UHPLC-HRMS)	5 *μ*g·kg^−1^	[14]

**Table 2:** Recovery and precision of three spiked levels.

Sample	Spiked concentration (*μ*g·L^−1^)	Recovery (%)	Intraday repeatability (%)	Interday reproducibility (%)
Apple juice	50	109.0	4.4	5.2
	200	94.3	2.3	4.8
	400	98.4	3.3	5.0
Ibuprofen	50	96.0	4.4	3.3
	200	100.9	2.5	7.0
	400	107.9	2.0	5.5
